# Practical Notes on Diagnosis and Treatment

**Published:** 1910-11-05

**Authors:** 


### PRACTICAL NOTES ON DIAGNOSIS AND TREATMENT.

### Chloral Hydrate.

THE taste of this can be well masked by ordering the syrup to be taken in effervescing lemonade.

### Gout.

IT is certain, first, that the peccant matter of gout is produced within the body, and is thus autotoxæmic, not entering infectively from without; and, secondly, that the subject of gout, either by inheritance or acquirement, is so far peculiar in his constitution that he reacts differently to various agencies, such as climate, food, etc., from persons not so disposed. The metabolic reactions of the gouty are abnormal. Many of the methods in practice relating to diet and therapeutics fail to regard the patient in a proper light, and all the efforts to set him right are directed against the perturbations of uric acid. This constitutes a radical fault in practice.—*Sir Dyce Duckworth.*

### The Renal Function in Urinary Disease.

THE symptoms of renal inadequacy do not always follow the variations of the mechanical defect in the bladder. They may be well marked with only an ounce or two of residual urine, and poorly developed with a fully distended bladder. In one hundred cases of stricture of the urethra, sixty-five had no symptoms of renal inadequacy. Of those with symptoms well marked some had strictures admitting large steel bougies. Pain was one of the most frequent symptoms of renal disease, and was situated, in the greatest number of cases, at the angle formed by the erector muscles with the last rib. The pain had usually a constant dull aching character.—*Mr. J. W. Thomson Walker.*

### Hypnotics.

NEVER allow a patient to know the name of the drug that he takes for the purpose of procuring sleep; it is by this means that we directly encourage the appalling habit of self-drugging that now threatens the very foundations of social life. Always use a drug with the whole action of which you are tolerably familiar. Personally, I am chary of going beyond opium and its derivatives or chloral, and I very much prefer to give a trial to simpler measures, such as sponging. I believe that, up to doses of ⅓ gr. in the case of morphia and 25 grs. in the case of chloral, these drugs are *not* depressants of the heart. As far as my experience goes there is no member of the coal tar series which has not a dangerously depressing effect upon the heart in certain people.—*Dr. F. J. Smith.*

### Migraine.

IN migraine there is undoubted evidence of abnormal vaso-motor action. Chilliness of the skin is common, and the temporal artery is hard and contracted. Relief of the pain often attends compression of the carotid artery. This and the fact that pain in the head is of a throbbing character suggest that the old explanation of the vaso-dilatation of the cerebral vessels is correct. The brain tissue itself is insensitive, and Harvey Cushing has noted that, after excision of the Gasserian ganglion, any subsequent headache is only felt on the side of the intact nerve—an observation which indicates that intra-cranial pain is experienced via branches of the fifth nerve to the membranes of the brain. Dilatation of the vessels with resultant increased tension in the dura would be enough to account for the pain in migraine.—*Dr. A. E. Russell.*

### Strabismus.

STATISTICS prove that most squints begin before or about three years of age, and unless training exercises are commenced before six years old the chances of overcoming the defects in the eye and brain are very remote. A concomitant squint is one in which the squinting eye can be moved freely in all directions, and besides the deviation of the optical axes there is also present a difference in the refraction of the two eyes and a deficiency in the "fusion faculty." In addition there is nearly always found a defect in the vision of one eye amounting to a loss of all useful vision, and an inability to bring the deviating eye to bear directly on any object.—*Mr. Bradburne.*

